# Does Early Mismatched Nutrition Predispose to Hypertension and Atherosclerosis, in Male Mice?

**DOI:** 10.1371/journal.pone.0012656

**Published:** 2010-09-09

**Authors:** Vanesa Bol, Fanny Desjardins, Brigitte Reusens, Jen-Luc Balligand, Claude Remacle

**Affiliations:** 1 Laboratory of Cell Biology, Institute of Life Science, Université Catholique de Louvain, Louvain-la-Neuve, Belgium; 2 Unit of Pharmacology and Theurapeutics, Université Catholique de Louvain, Brussels, Belgium; University of Camerino, Italy

## Abstract

**Background:**

A link between early mismatched nutritional environment and development of components of the metabolic syndrome later in life has been shown in epidemiological and animal data. The aim of this study was to investigate whether an early mismatched nutrition produced by catch-up growth after fetal protein restriction could induce the appearance of hypertension and/or atherosclerosis in adult male mice.

**Methodology/Principal Findings:**

Wild-type C57BL6/J or LDLr−/− dams were fed a low protein (LP) or a control (C) diet during gestation. Catch-up growth was induced in LP offspring by feeding dams with a control diet and by culling the litter to 4 pups against 8 in controls. At weaning, male mice were fed either standard chow or an obesogenic diet (OB), leading to 4 experimental groups. Blood pressure (BP) and heart rate (HR) were assessed in conscious unrestrained wild-type mice by telemetry. Atherosclerosis plaque area was measured in aortic root sections of LDLr−/− mice. We found that: (1) postnatal OB diet increased significantly BP (P<0.0001) and HR (P<0.008) in 3-month old OB-C and OB-LP offspring, respectively; (2) that maternal LP diet induced a significant higher BP (P<0.009) and HR (P<0.004) and (3) an altered circadian rhythm in addition to higher plasma corticosterone concentration in 9 months-old LP offspring; (4) that, although LP offspring showed higher plasma total cholesterol than control offspring, atherosclerosis assessed in aortic roots of 6-mo old mice featured increased plaque area due to OB feeding but not due to early mismatched nutrition.

**Conclusions/Significance:**

These results indicate a long-term effect of early mismatched nutrition on the appearance of hypertension independently of obesity, while no effect on atherosclerosis was noticed at this age.

## Introduction

Hypertension is estimated to affect 25% of the world adult population. It contributes to death from cardiovascular disease which remains a major cause of morbidity in the world [1] Hypertension coexists with other pathologies that are part of the metabolic syndrome. Although genetic and environmental factors are thought to play a role in these diseases, convincing evidence from human studies indicated that a suboptimal intrauterine environment, resulting in impaired fetal growth, is associated with an increased risk of developing insulin resistance, obesity, hypertension and cardiovascular disease in adult life. Seminal observations made by Barker and colleagues showed an increased death rate from ischemic heart disease, impaired glucose tolerance and type-2 diabetes in men with low birth weight [2], [3]. These observations gave rise to the “thrifty phenotype hypothesis” where the authors postulated that early malnutrition induces physiological and metabolic adaptations promoting early survival, which may however become deleterious for later health [4]. Since then, several animal studies have supported the concept that maternal malnutrition during pregnancy may exert long-term changes in the offspring [5], [6]. Recently, it has been suggested that the long-term effects of these adaptations to a suboptimal intrauterine environment might be more detrimental if there was a mismatch between individual's adaptations to the environment predicted to be experienced and the postnatal environment encountered. The predictive adaptive response (PAR) hypothesis was then formulated [7]. An example is provided by the comparison of the epidemiological data collected from two famines, in the North of Holland [8] and in Leningrad [9], [10]. Adult men that were submitted *in utero* to the Dutch famine in early pregnancy revealed more coronary heart disease, raised blood lipids, altered clotting and higher obesity compared to those who were not exposed, but such difference did not appear in the progeny analyzed in the Leningrad study. Whereas the Leningrad Siege lasted from 1941 to 1944, the Dutch famine lasted only for 6 months in a previously and subsequently well nourished population. This discrepancy between the two famines suggested that not only famine exposure, but also the rapid transition from suboptimal nutrition during pregnancy to adequate nutrition later on, could increase the risk for diseases in later life. Moreover, several studies indicated that rapid infant growth in humans is associated to an increased risk of presenting several components of the metabolic syndrome at adulthood [11] including hypertension [12].

Since then, new animal models of early mismatched environment have been established [13]–[15]. A recent publication by Cleal et al. [16] indicated that adult male sheep that had been underfed only during gestation and normally fed thereafter had altered cardiovascular function. The same was noted for sheep from the group of postnatal undernutrition alone but no alteration was observed in sheep that were constantly malnourished during gestation and lactation. Consequently, catch-up growth that was previously considered a process of recovery from detrimental effects of poor growth is now viewed as a risk factor for later health.

Boubred et al, 2009 [15] investigated in rat the effect of catch-up growth after protein restriction during gestation (LP) and found a lower nephron number in the LP offspring with or without catch-up growth that was accompanied by a transient hypertension in young age. They claimed that reduced nephron number associated with intrauterine growth retardation may not be sufficient to induce long-lasting hypertension and that a second hit such as early postnatal overfeeding is necessary. In their study, an exaggerated increase in visceral fat deposit was observed in the LP overfed group compared to the control overfed group. Previous work carried out in our laboratory also showed that adult male rats featuring a catch-up growth after fetal growth retardation induced by maternal protein restriction increased their susceptibility for obesity in adulthood [13], [14]. It is well known that obesity, together with a central pattern of fat distribution, represents a significant risk factor for hypertension and cardiovascular disease such as atherosclerosis. Indeed, adipose tissue is able to secrete a number of “adipokines” [17] some of them, like resistin, leptin, adiponectin, Monocyte Chemoattractant Protein-1, Interleukin-6, angiotensinogen, angiotensin-converting enzyme, plasminogen activator inhibitor-1 (PAI-1), being implicated in cardiovascular disease [rev. in 18, 19]. We have previously shown an increased expression of leptin, PAI-1 and angiotensinogen mRNA in offspring of dams underfed during gestation but only when catch-up growth had occurred [13], [14].

The aim of this study was to investigate whether a mismatch in early nutrition caused by catch-up growth after fetal protein restriction could induce hypertension and/or atherosclerosis in adult life and to assess if their development was obesity-dependent or not. As atherosclerosis does not develop spontaneously in rodents, several transgenic mouse models exist that allow study of atherogenesis when fed western-type diet [20]. We used LDL receptor deficient mouse (LDLr−/−), a well-characterized model.

Using these two types of experiment, we were able to show a long-term programming of early mismatched nutrition on the development of hypertension which is however independent of obesity, but we could not find an effect on atherogenesis.

## Methods

### Animals and experimental diets

To be able to reach the aim of this paper, 2 strains of mice with the same diet protocol were used. The C57BL6/J mouse strain (Janvier, Le Genest Saint Isle, France) was used for analyzing hypertension (Exp 1). Because normal mice do not spontaneously develop atherosclerosis, its programming was investigated in a strain which spontaneously develops such pathology, namely homozygous LDL-receptor knockout (LDLr−/−) mice (Jackson Laboratory, Bar Harbor, ME, USA) (Exp 2). Both strains were bred in our laboratory and maintained under controlled conditions (22°C, 12 h:12 h dark-light cycle). Non-primiparous females from both strains were housed with males overnight and mating was confirmed by the presence of a vaginal plug. The pregnant dams were housed individually during gestation and lactation. During gestation, dams of both strains were fed a control diet (C: 192 g protein/kg- 20% of energy) or an isocaloric low protein diet (LP: 81 g protein/kg- 8% of energy) purchased from Hope Farm (Woerden, Netherlands Hary block). The composition and source of diets were described elsewhere [21]. At birth, pups were weighed and litter size was recorded. In order to induce a catch-up growth, the number of pups was reduced to four in LP groups instead of eight in C litters. Moreover, during lactation, C and LP dams from both strains were fed with control diet. At weaning (4 weeks), in order to minimize the litter effect, only 2 male mice per litter were included in the study. For each litter, one male was fed standard lab chow (Carfil Quality, Turnhout, Belgium) and the other one was fed an obesogenic (OB) diet containing 16% sucrose, 18% lard, 27% casein and 23% rice starch (w/w); energy 4.56 kcal/g (Special Diet Services, Witham, UK). For each C57BL6/J cohort, half was analyzed at 3 months of age while the other half was followed until 9 months. The 2 (LDLr−/−) cohorts were analyzed at 6 months.

Body weight and food intake were recorded weekly after weaning in the C and LP groups of the 2 strains. At the end of experiments, mice received a lethal injection of Nembutal® (Natrii pentobarbitalum, 60 mg/kg bodyweight, CEVA, Brussels, Belgium). Different organs were dissected, weighed and stored at −80°C until analysis.

All LDLr−/− offspring were genotyped by PCR technique [22]. All procedures were carried out in accordance with “Principles of laboratory animal care” (NIH publication no. 85–23, revised 1985) and with the approval of the animal ethics committee of the Université catholique de Louvain, Belgium, Approval code 052803, to the laboratory LA1220028.

### (Exp 1) Circadian variation and frequency analysis of blood pressure and heart rate by implanted telemetry

Wild-type C57BL6/J male offspring were implanted with a telemetry device at 3 or 9 months of age because we aimed to verify if the early malnutrition followed by a rapid catch-up growth will precipitate the appearance of hypertension. Blood pressure catheters were surgically inserted in the carotid artery and miniaturized implants (TA11PA-C10, Data Science International Inc) placed in a subcutaneous pouch under anesthesia (ketamine/xylasine mixture). Recovery from the surgical procedure was estimated by body weight regain after 10 days. The mortality rates observed after telemetric implantation were <2%. Short- or long-term (24 hours) recordings were digitized (range 20 to 2000 Hz) and stored for further analysis. Short-term recordings were always performed between 10 to 12 a.m. In order to evaluate the blood vessels reactivity, the acute effect of L-NAME (30 mg/kg), an eNOS inhibitor, that induces an increase in BP by inhibition of NO production and activation of sympathetic activity and phenylephrine (100 µg/kg), a vasopressor alpha-1 adrenergic receptor agonist that also increases blood pressure were analyzed from short-term recordings after drug intra-peritoneal injection. Delta values of SBP and HR were calculated by subtracting the mean values of the 5 time-point after to the mean values of the 5 time-point before injection.

### (Exp 2) Analysis of atherosclerosis

Atherosclerosis plaque area was determined by analyzing cross sections from the aortic root of 6-mo LDLr−/− mice male offspring. After lethal injection, hearts were dissected, frozen in tissue freezing medium (Leica Microsystems, Nussloch, Germany) and stored at – 80°C until analysis. Approximately six 9-µm frozen sections per animal were used for morphometrical analysis and 6 aortic roots per group were analyzed following this procedure. Lipids were stained with oil red O and nuclei with hematoxylin. Blinded analysis of lipid stained area was performed with Axiovision quantification image analyzer (Axiovision Software 4.6.3, Carl Zeiss, Gottinghem, Germany) after capture of images with Axioscop 2 Mot Plus microscope (Carl Zeiss).

### (Exp 2) Real time RT-PCR

Total RNA was extracted from livers of LDLr−/− mice with Gen Elute™ Mammalian Total RNA miniprep kit (Sigma, St. Louis, MO, USA). The quality of RNA extracted from livers was assessed previous to reverse transcription by electrophoresis in 1% agarose gel and samples were stored at −80°C. First strand cDNA was generated from total RNA by RT using Ready-To-Go You-Prime first strand beads (GE Healthcare, Buckinghamshire, UK) following manufacturer's instructions. Quantitative real-time PCR was performed using SybrGreen master mix (Westburg Leusden The Netherlands). Sequences were designed using Primer Express software (Applied Biosystems, Foster City, CA, USA): TBP (NM_013684) 5′-TCCCAAGCGATTTGCTGC-3′ and 5′-GCAGTTGTCCGTGGCTCTCT-3′; PPARγ (NM_) 5′-TCTGGGAGATCCTCCTGTTGA-3′ and 5′-GAAGTTGGTGGGCCAGAATG-3′ and SREBP-1c (NM_011480) 5′- CGGCTGTCGTCTACCATAAGCT-3′ and 5′-CCAGTGTTGCCATGGATATAG-3′; HMG-CoASyn (NM_008256) 5′- TGCAGGAAACTTCGCTCACA-3′ and 5′- CCAGGTCAGTTTGGTCCACA-3′; HMG-CoARed (NM_ 008255) 5′- TCTTCCCGGCCTGTGTGT-3′ and 5′- CCTCACGGCTTTCACGAGAA-3′; SREBP-2 (NM_033218) 5′- GAGGTCACGAGGCTTTGCA-3′ and 5′- ACCGCTCCGCAGACGAGGA-3′. Oligonucleotides were purchased from Sigma. The level of mRNA expression was calculated using the ΔΔCt method were ΔCt of a sample represents the difference between Ct of target gene and the housekeeping gene and ΔΔCt is ΔCt of sample-ΔCt of calibrator consisting of cDNA from control mouse. The efficiency for housekeeping gene and each target gene was estimated in preliminary analyses and was assessed using standard curve method as explained previously [14]. The relative expression was calculated from 2^−ΔΔCt^ and expressed relatively to C where values are arbitrary set to 1. RNA levels were expressed as relative abundance ± SEM.

### Blood sampling and analysis

Blood was collected at sacrifice between 2 and 4 pm after 6 hours fasting by cardiac puncture in EDTA tubes and plasma was obtained by centrifugation. For glucose concentration analysis, proteins were precipitated by addition of 200 µl HClO_4_ (0.33N) to 20 µl blood and concentration was determined by the glucose oxidase method using Trinder's reagent (Stanbio Laboratory, Texas, USA). Triglyceride and total cholesterol concentrations were determined by using respectively TRF400CH and CTF400CH kits following the manufacturer instructions (Chema Diagnostica, Jesi, Italy). HDL-cholesterol concentration was assessed by the total cholesterol kit after precipitation of low-density and very low-density lipoproteins by “HDL precipitating reagent CD0400CH” (Chema Diagnostica, Jesi, Italy). Plasma insulin was measured using ultrasensitive insulin enzyme-linked assay (Mercodia, Uppsala, Sweden). Leptin concentration was determined by ELISA following manufacturer's instructions (Biovendor, Modrice, Czech Republic). Plasma corticosterone concentration was determined with an enzyme immunoassay kit (Assay Designs, Ann Harbor, MI, USA).

### Intraperitoneal Glucose Tolerance Test

Intraperitoneal glucose tolerance test (IPGTT) was performed after an overnight fast at 3 and 9 months in male offspring of C57BL6/J dams as well as at 6 months in male offspring of LDLr−/− dams. Glucose was measured after a single intraperitoneal bolus of glucose (20% glucose solution, 2 g/kg) in samples collected by tail bleeding after 0, 30, 60 and 120 minutes. Glucose concentration was determined with a glucometer (Ascencia Contour, Bayer Health Care, Mishawak, IN, USA).

### Statistical Analysis

Results are reported as mean ± S.E.M. Two-way analysis of variance was used to test for effects of maternal and post-weaning diets and interaction between these variables using the Prism Software (GraphPad Software Inc., San Diego, CA, USA). When specified, a Bonferroni post-hoc test was performed to look at statistical differences between groups. Differences with *P*<0.05 were considered as significant.

## Results

### Experiment 1: Programming of blood pressure and heart rate by mismatch of pre- and post-natal nutrition in C57BL6/J male offspring

#### Body weight and plasma parameters

As it has been shown in models of maternal protein restriction during gestation, pups from LP litters were growth restricted at birth when compared to C (C: 1.57±0.08 vs. LP: 1.21±0.03 g; N = 12 litters/group, P<0.001). As a consequence of forced catch-up growth during lactation, LP pups presented at weaning a higher body weight when compared to C (C: 10.94±0.82 vs. LP: 13.41±0.62 g; N = 12 litters/group, P<0.05).

**Figure 1:**
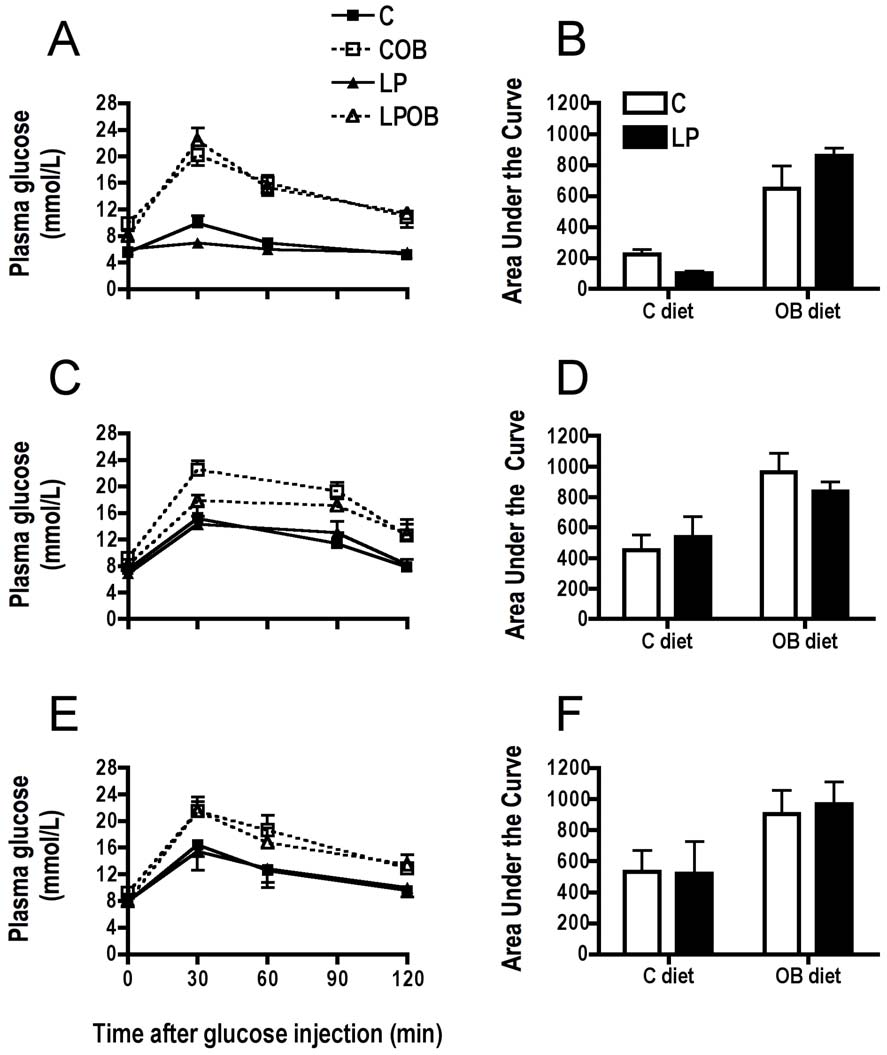
Blood glucose concentration and area under the curve (AUC) during intraperitoneal glucose tolerance test. The test was performed in 3-mo (A and B), 9-mo (C and D) C57BL6 male mice and 6-mo LDLr−/− (E and F). Values are presented as mean ± SEM with n = 5 to 8 individuals per group. Statistical analysis by Two-way ANOVA indicates a significant influence of post-weaning OB diet for AUCs at 3-mo (B; P<0.0001) and 9-mo (D; P<0.0001) in C57BL6 mice (*exp 1)* and at 6-mo (F; P<0.0001) in LDLr−/− mice (*exp 2)*.

At 3 months of age, both C and LP animals fed the OB diet after weaning presented a significant increase in body weight when compared to usual chow fed mice but no influence of maternal diet was found (Table 1). Analysis of plasma parameters indicated no difference for triglycerides or insulin concentration. However, the post-weaning OB diet increased glucose concentration in both the C and LP group but again no effect of the maternal diet was observed (Table 1). Moreover, intraperitoneal glucose tolerance test (IPGTT) indicated an altered glucose clearance in these OB fed animals as illustrated by the higher area under the curve (Figure 1A and B) but no effect of the maternal diet was noted.

**Table 1:** Body weight and Plasma parameters.

	Chow diet		OB diet		ANOVA		
***Experiment 1 (3-mo)***	**C (n = 6)**	**LP (n = 6)**	**C (n = 6)**	**LP (n = 6)**	*Interaction*	*Maternal diet*	*Adult diet e*
Body weight (g)	24.9±0.7	24.2±1.4	31.4±2.4	35.1±1.0	*NS*	*NS*	*<0.0001*
Glucose (mmol/l)	8.36±0.62	8.24±1.01	9.14±0.07	12.44±1.41	*NS*	*NS*	*0.0252*
Triglycerides (mmol/l)	0.52±0.09	0.62±0.05	0.75±0.12	0.65±0.06	*NS*	*NS*	*NS*
Insulin (ng/ml)	0.73±0.07	0.89±0.24	1.10±0.25	0.93±0.20	*NS*	*NS*	*NS*
***Experiment 1 (9-mo)***	**C (n = 5)**	**LP (n = 5)**	**C (n = 5)**	**LP (n = 5)**	*Interaction*	*Maternal diet*	*Adult diet e*
Body weight (g)	28.1±3.0	31.3±3.2	53.8±2.3	55.6±3.8	*NS*	*NS*	*<0.0001*
Glucose (mmol/l)	11.11±0.66	8.84±0.64	13.44±1.29	10.96±0.59	*NS*	*0.0106*	*0.0156*
Triglycerides (mmol/l)	0.38±0.02	0.55±0.08*	0.44±0.02	0.63±0.05	*NS*	*0.0077*	*NS*
Total cholesterol (mmol/l)	2.68±0.15	2.91±0.1	3.14±0.15	3.30±0.19	*NS*	*NS*	*0.0111*
Insulin (ng/ml)	1±0.5	0.8±0.2	4.9±0.4	5±0.3	*NS*	*NS*	*<0.0001*
Leptin (ng/ml)	0.9±0.1	2.0±0.6	17.4±2.6	18.0±2.7	*NS*	*NS*	*<0.0001*
Corticosterone (ng/ml)	32.7±8.2	52.9±9.6	36.1±11.8	93.4±25.6*	*NS*	*0.0234*	*NS*
***Experiment 2 (6-mo)***	**C (n = 8)**	**LP (n = 8)**	**C (n = 8)**	**LP (n = 8)**	*Interaction*	*Maternal diet*	*Adult diet*
Body weight (g)	28.2±0.9	29.1±0.9	40.9±2.0	43.9±2.8	*NS*	*NS*	*<0.0001*
Glucose (mmol/l)	9.89±0.36	9.69±0.54	11.22±0.52*	9.33±0.6	*NS*	*NS*	*NS*
Triglycerides (mmol/l)	1.04±0.11	0.71±0.06	1.93±0.31	3.20±1.01*	*NS*	*NS*	*0.0037*
Free Fatty Acids (mmol/l)	0.29±0.03	0.32±0.02	1.02±0.23	1.80±0.67	*NS*	*NS*	*0.0049*
Total cholesterol (mmol/l)	4.99±0.22	5.11±0.22	10.05±0.74	13.71±1.6*	*NS*	*0.0435*	*<0.0001*
HDL-Cholesterol (mmol/l)	0.85±0.06	0.83±0.05	1.21±0.06	1.38±0.09	*NS*	*NS*	*<0.0001*
Insulin (ng/ml)	1.3±0.2	1.5±0.2	3.7±0.3	4.6±0.3*	*NS*	*0.0421*	*<0.0001*
Leptin (ng/ml)	2.53±0.78	3.13±0.63	14.09±0.62	14.67±0.63	*NS*	*NS*	*<0.0001*

Data show mean ± SEM for n observations per group in the 2 experimental protocols at different time points. Difference between groups was analyzed using Two-way ANOVA (maternal diet, adult diet and interaction effect). P<0.05 was considered significant while NS stands for not significant. Bonferroni post-test has been performed to assess statistically significant difference within variables.

*P<0.05 compared to their respective control.

After 9 months, OB diet continued to induce a significant overweight in both C and LP groups, but no difference due to maternal diet was however observed. Plasma triglycerides concentration was significantly raised in LP animals when compared to C, showing an influence of maternal diet. Plasma glucose concentration was significantly decreased in LP group compared to the C group (Table 1). However, feeding mice offspring with OB diet increased glucose concentration in C and LP animals. Similarly, IPGTT indicated an altered response to glucose load in both OB groups as already observed at 3 months (Figure 1C and D). In addition, induced obesity was accompanied by hypercholesterolemia, hyperinsulinemia and hyperleptinemia in both groups. Maternal LP diet increased significantly the plasma corticosterone levels in LP offspring which was amplified by the OB post-weaning diet (Table 1).

### Circadian variation of blood pressure and heart rate

**Figure 2:**
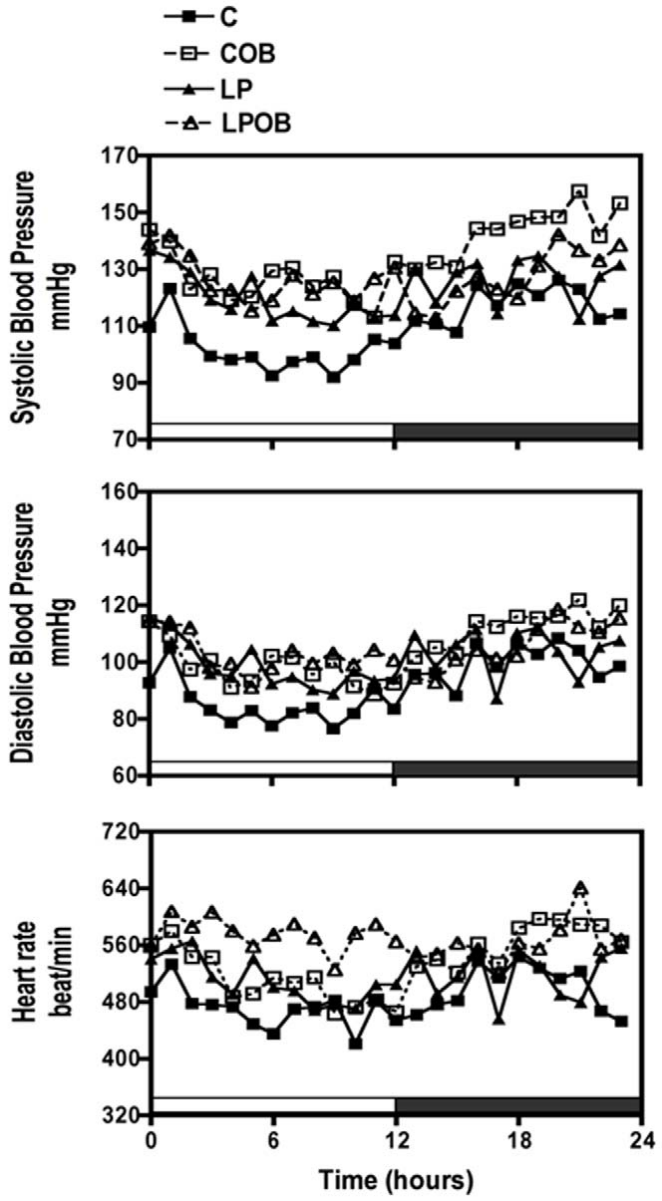
Long-term recording blood pressures and heart rate in 9-mo male C57BL6 mice (*exp1*). Recording of SBP (A), DBP (B) pressure and HR (C) were assessed during 24 h in n = 5 animals per group. Values are presented as mean values of SBP, DBP and HR calculated for each 60-minutes sequence of recording. Shaded zone on the X-axis indicates night period (activity period for mice).

Using telemetry, systolic blood pressure (SBP), diastolic blood pressure (DBP) and heart rate (HR) were continuously recorded during 24 h in 3- and 9-mo C57BL6/J male mice. At 3-mo we observed a slight but significant increase DBP and HR due to OB diet in C-OB and LP-OB groups during the day period. No difference was observed for these parameters during the night period. Maternal diet had however no effect at that age (Table 2). When these parameters were analyzed at 9-mo of age, a significant higher SBP and HR due to maternal diet was observed independently of obesity development (Figure 2 and Table 2). Statistical analysis of mean day and night parameters indicated a significant difference of SBP and HR only during day phase. Indeed, HR was significantly increased in the LP group under OB diet, and SBP was higher in the LP group under chow diet. As a consequence, LP animals lost the difference in blood pressure between day and night which normally occurs, reflecting therefore a loss of circadian rhythmicity in LP offspring (Table 2). Indeed, while C and C-OB mice showed a rise of blood pressure and heart rate during night phase, in the LP and LP-OB groups, blood pressure and heart rate remained constant along the 24 h period.

**Table 2:** Day and night average values of blood pressure and heart rate in 3- or 9-mo male mice.

		Chow diet		OB diet		ANOVA		
**3-months old** (n = 5)		**C**	**LP**	**C**	**LP**	Interaction	Maternal diet	Post-weaning diet
SBP (mmHg)	**DAY**	111±5	112±2	113±2	117±3	NS	NS	NS
	**NIGHT**	125±5	127±2	125±2	132±3	NS	NS	NS
DBP (mmHg)	**DAY**	90±4	87±4	94±2	98±3	NS	NS	**0.0251**
	**NIGHT**	103±5	100±4	102±2	112±3	NS	NS	NS
Mean HR (bpm)	**DAY**	490±12	471±16	499±11	527±10	NS	NS	**0.017**
	**NIGHT**	530±23	514±17	524±14	548±15	NS	NS	NS
		**Chow diet**		**OB diet**		**ANOVA**		
**9-months old** (n = 5)		**C**	**LP**	**C**	**LP**	Interaction	Maternal diet	Post-weaning diet
SBP (mmHg)	**DAY**	102±5	120±2*	142±7	126±9	**0.0076**	**0.0086**	**<0.0001**
	**NIGHT**	116±9	125±2	163±13	128±4	**0.0195**	NS	**0.0073**
DBP (mmHg)	**DAY**	85±6	99±4	99±6	103±2	NS	NS	NS
	**NIGHT**	99±11	103±2	111±8	105±5	NS	NS	NS
Mean HR (bpm)	**DAY**	472±28	511±16	513±28	577±5*	NS	**0.0039**	**0.0026**
	**NIGHT**	496±34	519±13	556±22	563±5	NS	NS	**0.0028**

Data are presented as mean ± SEM for n observations per group. Shaded values represent dark/night period (activity period in mice). The day averages were calculated as mean values from 9 to 20 h/individual, while dark period extends from 21 h to 8 h/individual. Difference between groups was analyzed using Two-way ANOVA (maternal diet, adult diet and interaction effect). P*<*0.05 was considered significant while NS stands for not significant. *Bonferroni* post-test has been performed to assess statistical significant difference within variables.

*P<0.05 compared to their respective control.

#### Short-term recording of SBP and HR after acute treatment by L-NAME or phenylephrine on 9-mo old mice

**Figure 3:**
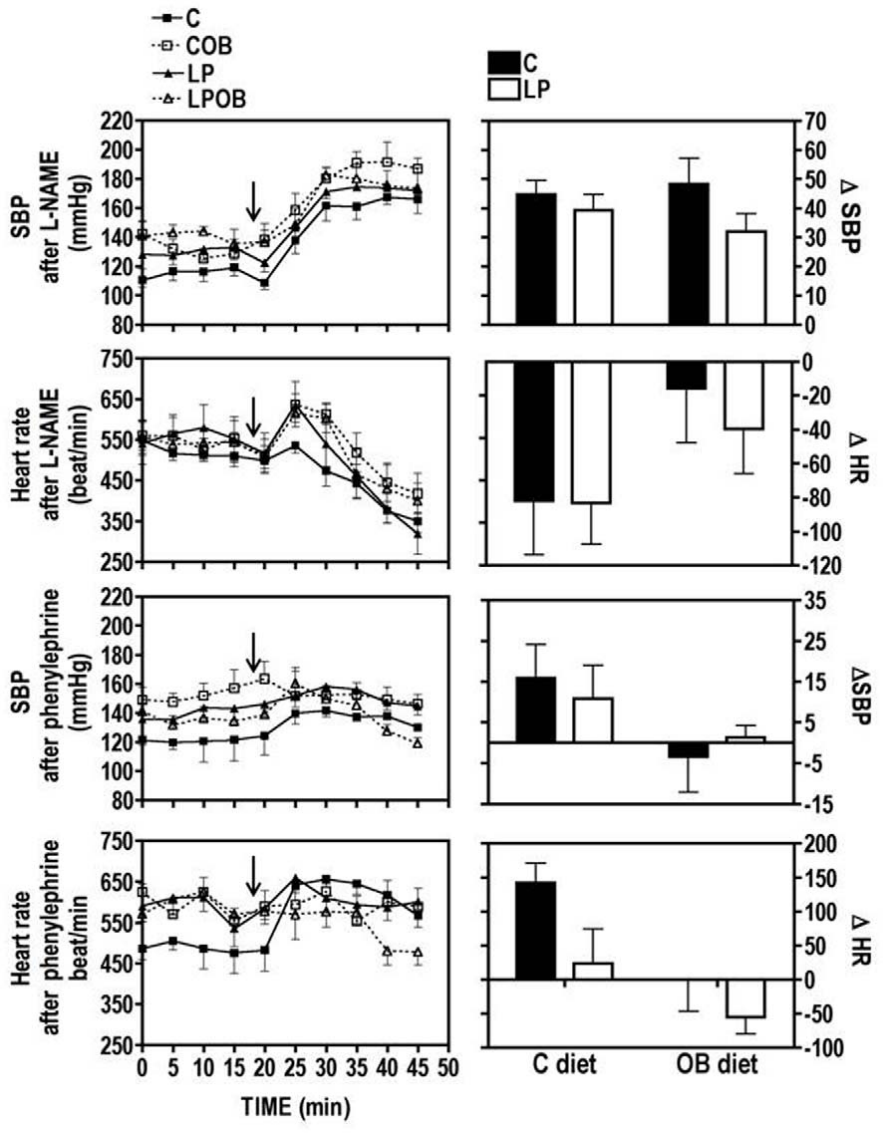
Short-term recording of SBP and HR after an acute treatment with L-NAME (A–D) and phenylephrine (E–H). Recording of cardiovascular parameters was assessed in 9-mo male C57BL6 mice (*exp1*) during 1 h in n = 5 animals per group. Values are presented as mean values of SBP or HR ± SEM calculated for each 5 minutes sequence of recording. Arrows indicate the time of injection. Δ values of SBP and HR were calculated by subtracting the mean values of the 5 time-point after to the mean values of the 5 time-point before injection. Statistical analysis by Two-way ANOVA indicates a significant influence of post-weaning OB diet (P = 0.0127) as well as a significant influence of maternal LP diet (P = 0.0429) for ΔHR after phenylephrine treatment.

Intraperitoneal injection with L-NAME (30 mg/kg), an e-NOS inhibitor that induces an increase in BP, produced a physiological response with a rapid enhancement of BP and a reduction in HR in the four experimental groups (Figure 3A). The magnitude of delta blood pressure reduction in mice from LP was not significant (Figure 3B). HR reduction tended to be lower in animals fed after weaning with OB diet (P = 0.07) (Figure 3C–D). As expected, the intraperitoneal injection of phenylephrine (100 µg/kg), an α1-adrenoceptor agonist that also increases blood pressure produced a slight increase in SBP but an important rise of HR in C animals (Figure 3E–F). When C and LP animals were fed the OB diet, they showed a minimal response to phenylephrine treatment either on SBP or HR, indicating a significant alteration of HR response due to OB diet (P = 0.01) (Figure 3G–H). Moreover, LP offspring displayed also an altered HR response to treatment indicating an influence of the maternal diet (P = 0.04) (Figure 3H).

### Experiment 2: Programming of atherosclerosis in LDLr−/− male mice by mismatch of pre- and post-natal nutrition

#### Body weight and plasma parameters

LDLr−/− pups from protein-restricted dams presented at birth a slight weight reduction that was however not significant (C: 1.37±0.04 vs. LP: 1.22±0.06 g; N = 8 litters/group). During lactation LP pups caught-up weight from day 14 and remained heavier than controls until weaning (C: 7.33±0.36 vs. LP: 8.62±0.16 g; N = 8 litters/group, P<0.001). After suckling period, OB fed animals gained rapidly more weight than chow fed mice and presented obesity when analyzed at 6-mo of age. Nevertheless, no influence of maternal diet on their weight gain was observed (Table 1).

Measurements of plasma parameters indicated a significantly higher concentration of triglycerides, free fatty acids and total cholesterol due to OB feeding. Total cholesterol was also increased due to early nutritional mismatch (Table 1) whereas HDL-cholesterol was increased in a similar way in C-OB and LP-OB animals when compared to chow fed animals. Although glucose concentration did not show any difference due to early nutrition or to OB diet, obese C and LP mice featured an altered response to glucose load as indicated by IPGTT (Figure 1E, F). Insulin concentration was raised in LP and LP-OB offspring as well as in C-OB fed animals compared to C offspring. Leptin concentration was also raised in obese mice without significant influence of maternal diet.

A comparison of body weight and blood parameters has also been done between the two strains (Experiment 1 and Experiment 2) and only few parameters showed a significant difference by strain. Body weight was significantly lower for LDLr −/− mice than for wild type mice when submitted to OB diet and might easily be explained by the age at which mice weight were recorded (9-mo vs. 6-mo). Not surprisingly, total cholesterol values for animals receiving OB diet were significantly higher for LDLr −/− when compared to wild type strain. Finally, plasma insulin values are statistically different in C-OB group between wild-type and KO mice and triglycerides values are significantly higher in LDLr −/− mice when compared to wild type, in LP-OB group.

#### Atherosclerosis measurements in aortic root of LDLr−/− mice and liver mRNA analysis with real-time PCR

**Figure 4:**
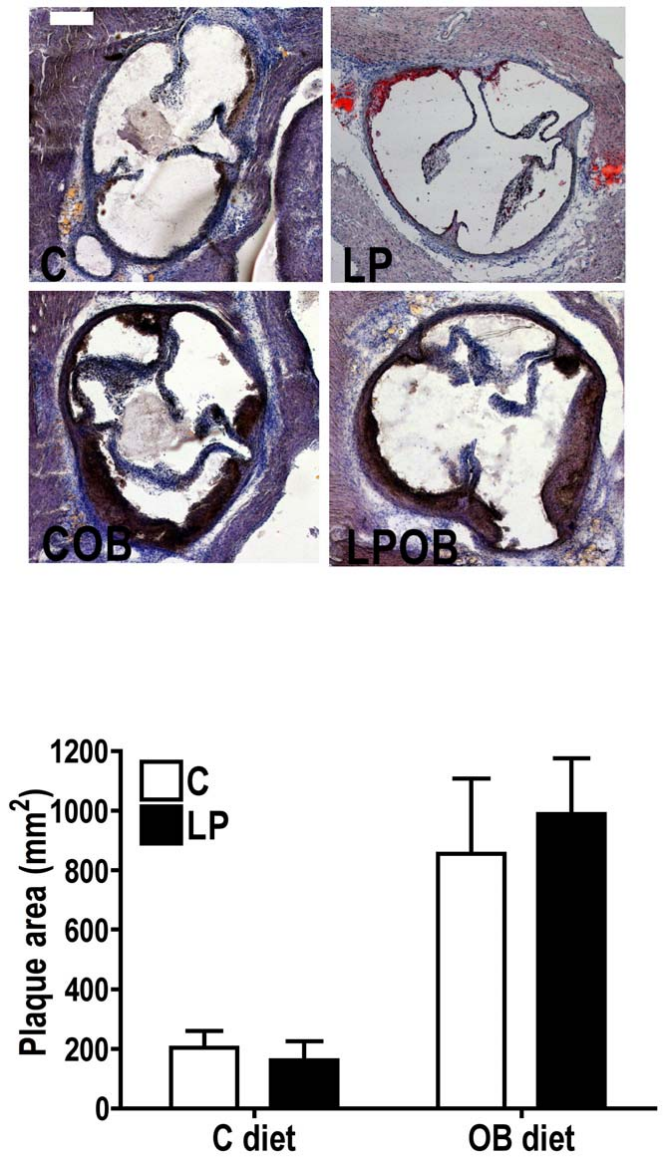
Atherosclerosis plaque area assessment in aortic root of 6-mo LDLr−/− male mice. (A) Representative images of oil red-O staining of the aortic root sections in the 4 experimental groups (scale bar = 200 µm). (B) The plaque area values are presented as mean values ± SEM for n = 6 animals per group. Two-way ANOVA indicates a significant influence of post-weaning OB diet (P = 0.0002) on plaque surface.

**Figure 5:**
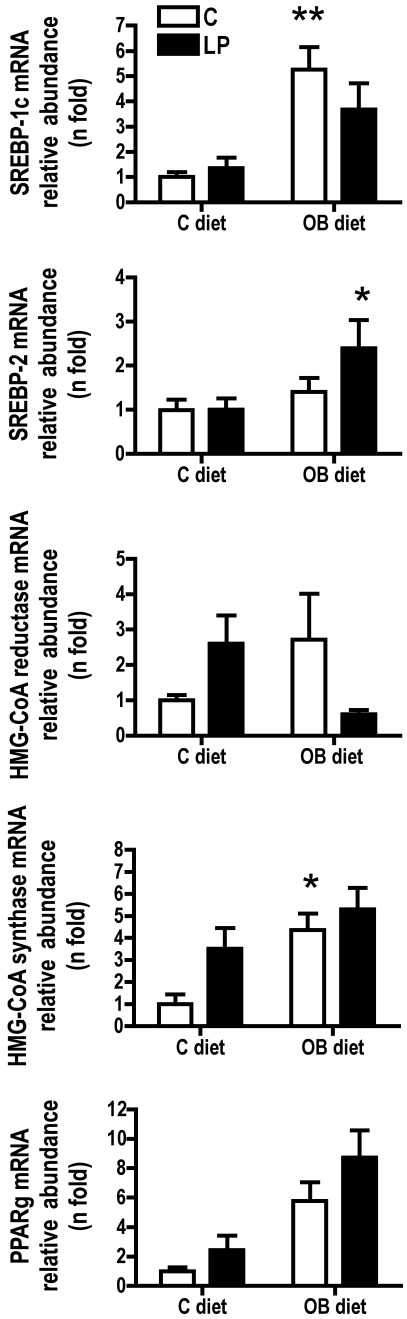
Relative abundance of gene mRNA assessed in livers of 6-mo LDLr−/− male mice. Values are presented as mean relative abundance ± SEM for n = 6 animals per group. Two-way ANOVA indicated a significant influence of post weaning OB diet for SREBP-1c (P = 0.0002), HMG-CoA synthase (P = 0.004) and PPARγ (P = 0.0002) as well as a significant influence of maternal LP diet for SREBP-2 (P = 0.034) and HMG-CoA synthase (P = 0.0406). HMG-CoA reductase showed a significant interaction effect (P = 0.0243). Bonferroni post-test has been performed to assess statistical differences in mRNA relative abundance within chow fed vs. OB fed animals within C and LP groups. * P<0.05 and ** P<0.01.

Figure 4A shows representative oil red-O stained sections of the aortic root from male mice of the 4 experimental groups. Measurements of lesion area indicated an increase of plaque surface in both OB fed animals (P = 0.0002; Figure 4B). No difference according to maternal diet was observed in this experiment. Nevertheless, since plasma total cholesterol was higher in LP and LP-OB offspring, we investigated the mRNA expression of few liver genes involved in cholesterol metabolism. PPARγ and SREBP-1c mRNA levels were both significantly increased according to post-weaning OB diet (P = 0.0002; Figure 5). Although the expression of PPARγ was higher in LP groups when compared to C groups, the difference was not significant. HMG-CoA reductase mRNA abundance showed a significant interaction between the maternal diet and OB diet effect meaning that the effect of OB feeding was different according to maternal diet (P = 0.0243) while HMG-CoA synthase expression showed a significant effect of OB feeding (P = 0.004) as well as a significant effect of maternal nutrition (P = 0.0406). Finally SREBP-2 mRNA relative abundance showed an increase due to OB diet (P = 0.034). However, the increase was more important in LP-OB group than in C-OB as indicated by significant Bonferroni post-test (Figure 5).

## Discussion

This study aimed to determine whether an *in utero* growth retardation followed by a rapid catch-up growth after birth was able to favour hypertension and atherosclerosis at adult age, and, if this was dependent on obesity. Two main results may be pointed out in the first experiment where hypertension was analyzed. Firstly, at 9-mo LP offspring presented higher SBP and HR than C offspring, indicating a programming of cardiovascular function by the poor prenatal diet followed by a rapid catch-up growth. In addition, this programming is independent of obesity development because hypertension was already observed in LP offspring fed a normal diet. A second important finding is the loss of circadian rhythms of the blood pressure and heart rate in the LP offspring.

Early postnatal overfeeding is known to induce cardiovascular and metabolic changes such as hypertension and central obesity with increased leptin, glucose, insulin, and glucocorticoid level [23]. In addition to intrauterine growth retardation, this may be a second hit which favors hypertension and cardiovascular disease [15]. Consistent with this, in our study, overweight resulting from post-weaning high fat diet induced an altered glucose tolerance in both OB fed groups at the age of 3-mo and these animals presented a rise in DBP and HR during the resting (day) period (Exp 1). However, contrary to what has been shown by others [15], [24], [25] no effect due to maternal diet was observed at this young age either on BP or HR. Higher SBP and HR were only revealed with age in LP offspring after 9-months. In a rat study, when the LP diet was given during gestation and maintained throughout life, catch-up growth did not occur and blood pressure as well as renal glomerular structure remained unaltered [26]. Whereas some studies using tail cuff technique depicted a rise of BP due to the maternal protein restriction during pregnancy in 3-mo rats [13], [24], [25] another group using telemetry recording showed only moderate effect of a more severe maternal protein deprivation on hypertension in young offspring [27]. This puts forwards the impact of the technique used, as data obtained by tail-cuff method are based on indirect blood pressure measurements which require restraint, a potential source of stress, and it is know that the LP offspring are more susceptible to stress at least in female offspring [28].

Many bodily functions such as temperature, eating, blood pressure and heart rate are influenced by circadian clock. Blood pressure in rodents, as in human, presents a circadian pattern and its loss correlates with a higher risk for cardiovascular complications [29], [30]. One of the factors that limit the study of cardiovascular function is the difficulty to measure blood pressure in conscious non-stressed animals. Indeed, the measurement of the basal levels and the circadian patterns is not possible using the tail cuff technique. Here we have used the gold standard technique of implanted telemetry to investigate this function. Our results highlighted a programming of alteration in the circadian clock by the early diet that may have influenced the blood pressure. In the control groups, we found a greater SBP, DBP as well as HR during the night (activity period) than during the day which represents a normal pattern [31]. This was not found in our LP groups and the HR was even higher during the day than during the night (under OB diet). Thus, LP male offspring featured a loss of circadian rhythms of both blood pressure and heart rate. Very recently, the study of Samuelsson et al [32] revealed that maternal diet-induced-obesity was able to program hypertension in male and female offspring at 6 months but only the male offspring of the obese mother lost the diurnal blood pressure variation.

It was postulated that the functional significance of the normal increase in blood pressure and heart rate during the active period, which is during the dark phase in rodent, is to allow the animals to meet the metabolic demands of increased activity [31]. In obese Zucker rat circadian disturbance of activity and feeding occurs with enhanced food-anticipatory activity [33], [34]. Programming of dysregulation in the day-light behaviour was also observed for food intake rhythm in rats after a drastic maternal food restriction during gestation. In such condition, a decreased food intake in the dark phase and an increased food intake during the light phase compared to control group were reported [35]. Another recent study evaluated the consequence of an early or late catch up-growth after IUGR due to maternal protein restriction on the feeding behaviour during day and night phase and found that more than the quantity of food ingested, it was the duration of meals that was modified by the nutritional programming [36]. In this model, the early catch-up growth reduced the abnormal organization of hypothalamic pathways involved in energy homeostasis [37]. Our data together with those reported above open new perspectives for investigating another pathway involved in early programming because the disturbance of the circadian rhythmicity may influence food behaviour, obesity, hypertension and other diseases which have been found to have early origin.

The molecular clock in mammals is located in the suprachiasmatic nucleus (SCN). However there are molecular clocks in many peripheral tissues as in the liver, kidneys and heart [38]. Even if mechanisms linking the SCN and peripheral clocks are still poorly understood, they might involve circulating hormones [39]. Recent results suggest that the rhythmic secretion and the ability to phase shift peripheral clocks place glucocorticoids as possible candidates for the entrainment of peripheral oscillators [40] and interestingly plasma corticosterone levels were increased in the LP offspring.

On the other hand, HR and BP are under the control of different factors among which nitric oxide and several hormones. Therefore, we investigated BP and HR after an acute treatment with L-NAME, whose vasoconstrictive effects are directly modulated by the endothelium and with phenylephrine, an alpha1-adrenergic vasoconstrictor. Little change in the vascular reactivity was observed in relation to the maternal diet. Indeed, only HR showed an altered response due to maternal LP diet after phenylephrine injection. Sathishkumar et al [41] investigated the blood pressure and the vascular reactivity in 3-month old female offspring from mothers fed a low protein diet during gestation and showed an increased BP pressure and contractile response to phenylephrine, as well as, a reduced vasodilatation in response to acetylcholine. It should be noted that in this study, no postnatal catch-up growth occurred and the LP offspring remained lighter throughout life. Glucocorticoid plasma concentration which was increased in LP offspring is thought to play a main role in the early programming of adult disease. In rodents, fetal exposure to excess glucocorticoids via maternal stress (calorie restriction) or drug treatment (dexamethasone) lowered birth weight, increased glucose and insulin levels and also increased blood pressure in the adult progeny [42]. Correlatively, treatment of LP mothers during pregnancy with metyrapone, an inhibitor of corticoids synthesis, prevented the development of high blood pressure in male offspring [43]. Other studies identified increased adrenal-to-body weight ratio in parallel to an increased resting heart rate [44] and higher levels of peripheral epinephrine levels in LP male offspring, as well as perturbation of β-adrenergic response and signaling [45]. Finally, it was found that prenatal dexamethasone decreased basal blood pressure but induced stress-hypertension in adult rat offspring [46]. In parallel to our observations, these reports indicate a link between programming of hypertension and stress-response axis. Indeed, it is thought that fetal environment could program an enhanced activity of the mediators of stress response including hypothalamic-pituitary-adrenal (HPA) axis and autonomic nervous system [47]. A suboptimal *in utero* environment could alter the set points of such stress axes in order to provide a biological advantage. However, if the environment encountered later on is not well matched to the fetus predictions, it could provide a disadvantage and lead to hypertension later in life. Although in our experiments, an acute treatment with phenylephrine rapidly increases SBP in C animals, only a slight increase was observed for LP offspring. We postulate that a permanent elevated level in stress hormone for these animals may reduce the response to the acute treatment with the α1-adrenoreceptor agonist.

In the second experiment, we used the well-characterized LDLr-/- mice to study the impact of a mismatched early nutrition on the development of atherosclerosis. Although we were unable to show an influence due to maternal diet, feeding animals after weaning with OB diet increased the atherosclerosis plaques extent. Only a few papers examined the influence of early diet on the development of atherosclerosis in transgenic mice models. These studies mainly focused on the impact of maternal hypercholesterolemia for the programming of atherosclerosis. In LDL receptor knock-out (LDLr−/−) mouse, as well as, in ApoE heterozygous deficient mouse, maternal hypercholesterolemia -induced either by diet or by cross-breeding wild type male mouse with ApoE deficient females- promoted atherosclerosis in aortic arch of the respective offspring [48], [49]. Another recent study has investigated the programming of atherosclerosis by fetal malnutrition in ApoE*3 Leiden mouse [50]. Such mice have an impaired clearance of lipoproteins from the plasma, a raised plasma lipid levels and a greater susceptibility to develop atherosclerosis when the mice are fed diets rich in cholesterol [51]. No change in cholesterol level and atherosclerosis was observed when the offspring were fed a normal diet. The post-weaning atherogenic diet induced however a higher level of cholesterol and a greater area of atherosclerotic lesions within the aortic arch in animals exposed to a low protein diet *in utero* than those exposed to the control diet, but this was noted only in females [50]. In our study, although no difference in atherosclerosis development was observed due to early mismatched nutritional environment, plasma total cholesterol concentration was however increased due to maternal diet. Moreover, the analysis of plasma HDL-cholesterol concentration showed a similar increase for animals of the C-OB and LP-OB group indicating that the higher values of total cholesterol observed in LP-OB mice were due to increased amount of LDL- and VLDL-cholesterol. Therefore we also investigated the mRNA expression of genes involved in liver cholesterol biosynthesis. The results indicated an increase due to OB feeding for almost all genes. However, the expression of HMG-CoA reductase, the rate limiting enzyme required for endogenous cholesterol biosynthesis was lower in LP-OB group when compared to C-OB group while conversely; it was increased in the LP group compared to the C group. It indicates that the higher concentration of circulating total cholesterol observed in LP-OB offspring is not due to an enhanced biosynthesis.

It has been shown that plasma total cholesterol tended to be higher in 18-mo rats that were protein restricted only during fetal life [52] while another experiment in similar conditions indicated a lower plasma cholesterol for these animals at 6-mo [53]. A possible explanation for the differences observed in these studies is the establishment of an insulin resistant phenotype that develops with aging [54]. In our study, animals from the LP-OB group were fed with an obesogenic diet after weaning which worsened the insulin resistant state as shown by their plasma insulin levels and their IPGTT. In addition, it has been recently demonstrated that mice with diabetes and that lack LDL receptor develop severe hyperlipidemia with decreased clearance in lipoproteins [55]. Finally, statistical analysis of body weight and plasma parameters between both strains (experiment 1 and 2), demonstrated consistency in the maternal LP model since no main influence of the strain was observed for comparable plasma parameters, except for blood lipids, as expected.

In conclusion, our results showed a long-term effect of early mismatched nutritional environment on the development of hypertension independently of obesity. Moreover, 24 h recording of blood pressure and heart rate indicated impairment in circadian rhythms for animals that were exposed *in utero* to LP diet and forced to catch-up growth during lactation. Although no influence due to maternal diet was observed, atherogenesis was enhanced by postnatal OB diet. Finally, even if there is now convincing evidence from epidemiological and animal studies, supported by our results, that early environmental mismatch can lead in adulthood to physiological changes, the mechanisms underlying these long-term alterations are not yet understood. Research needs to be carried on further and identification of key players should be important for prevention of metabolic diseases in adulthood.
